# Pancytopenia with severe thrombocytopenia in asymptomatic malaria in advanced pregnancy: a case report

**DOI:** 10.11604/pamj.2022.41.154.30168

**Published:** 2022-02-22

**Authors:** Grant Murewanhema, Tapuwa Carol Musiniwa, Maxwell Takura Chimhina, Simbarashe Madombi, Munyaradzi Innocent Nyakanda, Mugove Gerald Madziyire

**Affiliations:** 1 Unit of Obstetrics and Gynaecology, Department of Primary Health Care Sciences, Faculty of Medicine and Health Sciences, University of Zimbabwe, Harare, Zimbabwe,; 2 Parirenyatwa Group of Hospitals, Ministry of Health and Child Care, Harare, Zimbabwe,; 3 Zimbabwe Society of Obstetricians and Gynaecologists, Harare, Zimbabwe,; 4 Sally Mugabe Hospital, Ministry of Health and Child Care, Harare, Zimbabwe

**Keywords:** Zimbabwe, malaria in pregnancy, pancytopenia, thrombocytopenia, case report

## Abstract

Malaria in pregnancy is associated with significant morbidity and mortality, and requires early diagnosis and intervention. Plasmodium falciparum is responsible for 98% of malaria cases in Zimbabwe and causes the most severe disease. Abnormal haematological parameters are a frequent finding in patients with malaria; however, they are rarely the sole presenting feature. We present the case of a 32-year-old woman in her fifth pregnancy, with a history of one previous caesarean section, who presented for caesarean section and was incidentally noted to have severe thrombocytopenia. Subsequent investigations at a tertiary institution revealed a pancytopenia with thrombocytopenia as the most prominent feature in an asymptomatic patient. The unavoidable caesarean section done under platelet cover was eventful, with severe intractable haemorrhage necessitating an emergency hysterectomy. However, the patient made a full recovery with antimalarial treatment and blood product transfusions. This case is presented to illustrate the need to consider malaria as a differential diagnosis in pregnant patients from malaria-transmitting areas who have thrombocytopenia. Previous studies have shown that thrombocytopenia can be a predictor of malaria in patients who present with fever, and a marker of disease severity, but has no utility in prognostication and follow-up.

## Introduction

This case is reported according to the CARE guidelines [1]. Estimates indicate that malaria results in over 10,000 maternal deaths annually across the globe, the majority of which occur in sub-Saharan Africa [2,3]. In the Zimbabwe maternal and perinatal mortality survey, malaria was one of the leading indirect causes of maternal mortality (MM), accounting for 7.4% of the notified deaths [4]. *Plasmodium falciparum* causes the most severe malaria, and accounts for over 98% of the cases [5]. Pregnant mothers are particularly susceptible to developing severe malaria [6,7], owing to suppression of cell-mediated immunity and preferential sequestration of parasites in the placenta [2,3,8]. Malaria in pregnancy is associated with significant pregnancy wastage, anaemia, intrauterine growth restriction and low birth weight babies [3,9]. Primigravidae are more susceptible to complications of malaria than multigravidae [2]. The case fatality rate of malaria in pregnancy can be as high as 50% [3], considerably higher than in the non-pregnant women population. Thus, intermittent preventive therapy (IPT) is advised for women residing in endemic areas [10-12]. The presentation can be atypical in women residing in malaria-endemic areas, who have a level of background immunity [9].

Malaria is a haematological disease, with anaemia and thrombocytopenia as frequent features [13,14]. However, presentation with severe thrombocytopenia as the first suggestive feature without fever or other symptoms is infrequent. Several patho-mechanisms with biological plausibility for thrombocytopenia include coagulation disturbances, bone marrow alterations, splenomegaly, antibody-mediated platelet destruction, oxidative stress and the role of platelets as cofactors in triggering severe malaria [15]. We present a case of *P. falciparum* malaria in advanced gestation, which was only suspected and diagnosed because of severe thrombocytopenia noted when patient was being prepared for caesarean section (C/S). We present the case to illustrate the possibility of missing such a potentially fatal diagnosis, and the need for continued vigilance by attending clinicians.

## Patient and observation

**Patient information:** a 32-year-old woman in her fifth pregnancy with three children alive and one first trimester miscarriage was referred to our hospital, a tertiary institution, from a provincial hospital. She had a previous C/S, and had presented late at an estimated menstrual gestational age of 42 completed weeks and four days. On work-up for C/S at the referring hospital, she was noted to have a severe thrombocytopenia of 34x10^3^/µL. The reasons for referral were the absence of blood products and appropriately qualified anaesthesiologists at that hospital. On systemic enquiry at presentation, she reported generalised body weakness in the preceding three-to-four days that she attributed to advanced pregnancy and did not bother to seek medical attention. She had no fever, chills and rigors, joint pains nor headaches, and the rest of her systemic enquiry was non-contributory. The pregnancy was booked, and she had six antenatal care (ANC) visits. She was HIV negative, and had no other chronic illnesses. No ultrasound scan (USS) was done due to financial constraints. She was on hematinic supplements from the booking visit; however, she did not receive IPT for malaria, and did not take any other medicines. In the first trimester, she was treated for slide-positive symptomatic malaria with a complete course of co-artemether (artemether/lumefantrine). She had a history of one C/S performed for fetal distress in her last pregnancy in 2015, after two vaginal deliveries and a first trimester miscarriage. She stayed with her family in Burma Valley, which is malaria-endemic.

**Clinical findings:** she looked rather unwell, pale and dehydrated, with no jaundice or lymphadenopathy. The blood pressure was 110/56 mmHg with a heart rate of 119 beats per minute, respiratory rate of 14 breaths per minute and a temperature of 36.5 °C. Chest, cardiovascular, central nervous system and skin examinations were unremarkable, and she did not have epigastric tenderness on abdominal examination. She had a Pfannenstiel scar. The symphysis-fundal (SFH) was 38 cm, with a singleton foetus in longitudinal lie and cephalic presentation. The foetal heart rate was 132 beats per minute.

**Diagnostic assessment:** the problems noted were the severe thrombocytopenia, one previous C/S and post-term pregnancy. Laboratory investigations revealed a pancytopenia, and numerous ring forms of *P. falciparum*. However, no level of parasitemia was provided. The white cell count was 2.0x10^3^/µL, haemoglobin level was 9.8 g/dl and platelet count was 29x10^3^/µL, with a mean corpuscular volume of 81.1 fL. The urea, electrolytes and liver enzymes were all normal with an alanine transaminase (ALT) of 13 iU/l and an aspartate transaminase (AST) of 36 iU/l. Both total and direct bilirubin were within normal limits. Bedside clotting time was prolonged at 13 minutes. Despite low normal BPs, urinalysis was done and was negative for proteins.

**Therapeutic intervention:** diagnoses of *P. falciparum* malaria, one previous C/S and post-term pregnancy were made. Treatment for malaria with intravenous (IV) artesunate 2.4 mg/kg body weight once daily, ceftriaxone 1 g IV once daily, and paracetamol 1 g IV three times a day was commenced. An emergency C/S was done under platelet cover and tranexamic acid 1 g infusion, and a live baby weighing 3195 g with Apgar scores of 8/10-9/10 was delivered. However, post C/S the patient was noted to be actively bleeding per vagina, and a re-laparotomy was done, proceeding to a supra-cervical hysterectomy after she had continued bleeding despite uterotonics, traexamic acid infusion and uterine massage. The total estimated blood loss was 2500 ml, and the patient was transfused three units of packed cells, 6 units of platelets and 6 units of fresh frozen plasma.

**Follow-up and outcomes:** post-operatively, the patient remained anaemic, with a haemoglobin level of 7 g/dl, and she was transfused two more units of packed cells. She had a good urine output of 1800 mls of clear urine in the first 24 hours, and started mobilising on the first day. The repeat malaria thin slide was negative, and she was discharged on co-artemether (artemether/lumefantrine), paracetamol and oral antibiotics on the fifth day. For further postnatal follow-up, she was referred back to the referring hospital to minimise the costs and need for travelling.

**Patient´s perspective:** the patient was very grateful that both her and the baby were well on discharge, and was also grateful that she had been diagnosed of malaria before she had developed fatal complications.

**Informed consent:** the patient provided signed written informed consent for case publication. However, the Medical Research Council of Zimbabwe and the Joint Research Ethics Committee do not require approvals for case reports.

## Discussion

We have presented a maternal near-miss case due to malaria-induced haematological dysfunction in a patient who had no overt malaria symptoms. A history of residence in an endemic area, previous treatment of malaria in current pregnancy and a severe isolated thrombocytopenia prompted investigations for malaria. Recrudescence and recurrence of malaria after treatment have been reported, regardless of treatment regimen. In a retrospective analysis, Laochan *et al*. noted 909 recurrent episodes of *P. falciparum* malaria out of 700 pregnant women treated with several regimens in Thailand, with 481 novel infections, and 428 recrudescent infections, confirmed by polymerase chain reaction (PCR) genotyping [16]. Periods of recrudescence varied from 28 days up to greater than 100 days, with the longest periods reported after the use of artemisinin-based combination treatments (ACT) [16]. Comparable findings were noted in a trial in Zambia, where more recurrent infections were noted with artemether-lumefantrine combinations compared to mefloquine-artesunate or dihydroartemisinin-piperaquine combinations [17]. Our patient received a complete course of artemether/lumefantrine and had a recrudescence months later.

An unavoidable C/S was done in a patient with a previous C/S and a post-term pregnancy under adequate platelet cover, and with the administration of the antifibrinolytic tranexamic acid. It is recognised that patients with thrombocytopenia at surgery may bleed despite platelet cover, and a pro-active approach to minimise bleeding was undertaken, hence the prophylactic administration of tranexamic acid. Thrombocytopenia is well-recognised complication of *P. falciparum* malaria. In some reports, the frequency is 24-94% in patients with acute malaria [15]. It rarely is the sole presenting feature. Clinicians operating in malaria-transmitting countries must always keep malaria on the list of differentials; however, atypical cases may present diagnostic delays. Our patient was referred with thrombocytopenia; however, a repeat evaluation revealed a pancytopenia, with leucopenia and anaemia. Hobeika *et al*. reported a case of a primigravida who had pancytopenia and hepatosplenomegaly without fever at 26 weeks of gestation [18]. Due to absence of suggestive clinical features there was delayed diagnosis, she was transfused 10 times, had a splenectomy and malaria was only diagnosed two months after splenectomy [18].

Thrombocytopenia is the second most common haematological derangement after anaemia in pregnancy and the reported frequency is 6.6-11.2% [19]. Several aetiologies, pregnancy or non-pregnancy related exist, which can be congenital or acquired. Diagnostic algorithms may help elucidate the causes. Important differential diagnoses include pre-eclampsia, HELLP syndrome, acute fatty liver of pregnancy (AFLP), thrombotic thrombocytopenic purpura (TTP), autoimmune thrombocytopenia and haemolytic-uremic syndrome (HUS) [19,20]. In these pathologies, thrombocytopenia occurs as part of a spectrum of clinical signs and symptoms, and does not usually occur alone except in autoimmune thrombocytopenia. Patients with TTP are very sick, with neurological manifestations. HUS is a serious disease, with renal complications that typically is preceded by a diarrhoea illness but sometimes can be atypical, preceded by urinary tract or other infections. Gestational thrombocytopenia occurs in 5-8% of pregnancies, and is responsible for more than 70% of maternal thrombocytopenia [20]. However, the platelet counts rarely fall below 100x10^3^/µL, and the condition is usually clinically inconsequential.

Evidently, malaria is a haematological disease, with frequent severe anaemia, coagulopathies, leucocyte numerical or functional changes and spleen involvement [13]. In a cross-sectional study by Awoke and Arota to profile haematological parameters in *P. falciparum* and *P. vivax* malaria at a hospital in South Ethiopia, thrombocytopenia and anaemia were noted in 84% and 67% of malaria patients respectively [21]. An inverse correlation was noted between parasitemia level and lymphocyte and platelet counts. In another hospital-based cross-sectional study in a region of Ghana, red cell and platelet counts were significantly lower in malaria than non-malaria infected patients [14]. Ndamukong-Nyanga *et al*. noted similar findings in another cross-sectional study in Buea, Cameroon [22]. They noted thrombocytopenia and anaemia to be the most significant predictors of malaria in their study region and concluded that these parameters could improve malaria diagnosis when utilised in conjunction with other clinical features. Notably, the biggest weakness of all these studies is that they were cross-sectional studies, subject to reverse-causality. Longitudinal studies with a follow-up component may be a better design to study this important area.

Pancytopenia, like in the case we presented, has also been observed frequently. Sakzabre *et al*. in a retrospective analysis in Ghana, observed lymphopenia (56.78%), anaemia (55.51%), thrombocytopenia (47.46%), eosinopenia (45.76%), neutropenia (29.24%), monocytosis (21.19%), and leucocytosis (17.37%) in infected patients [23]. However, they had a significant number of cases due to *P. malariae* (12.7%), and this may have altered the clinical picture. Nevertheless, the study adds to the pool of evidence that malaria is a haematological disease. Even studies beyond Africa corroborate these findings. Lopez-Perez *et al*. conducted an observational study in Colombia, Latin America in a low-malaria transmitting area and observed mild-to-moderate anaemia in 68% of their study participants, and thrombocytopenia in 41% of their study participants [7]. *P. vivax* infections however, are more prevalent in this region, compared to Africa where *P. falciparum* accounts for the majority of the cases, hence the results may not be directly comparable [7].

Studies have been carried out to evaluate the predictive value of thrombocytopenia for malaria. In a longitudinal study of 228 patients who presented to hospital with fever and thrombocytopenia, 53% tested positive for malaria (68% *P. falciparum* and 32% *P. vivax*) [24]. In malaria transmitting regions, the presence of fever and thrombocytopenia can thus be a predictor of malaria infection. Hanson *et al*. noted that thrombocytopenia is a marker of disease severity in *P. falciparum* malaria, though its value in prognostication, triage and management is limited [25]. There is generally no specific treatment for thrombocytopenia in malaria, which tends to recover as the disease process improves with treatment. Tan *et al*. in their study noted the median time for recovery from malaria associated thrombocytopaenia to be 7 (range 2-14) days and did not differ by treatment regimen [26]. However, patients requiring surgery and those who develop bleeding disorders may need supportive platelet transfusions.

## Conclusion

Pregnant women from malaria-transmitting areas who have thrombocytopenia must be routinely investigated for malaria. Thrombocytopenia is a predictor of malaria, and can be a marker of disease severity in patients with *P. falciparum* malaria. However, attending clinicians must also rule out other causes of thrombocytopenia in pregnancy, corroborating clinical history, findings and diagnostic evaluations, as there are other causes of thrombocytopenia in pregnancy, which are also quite as fatal. Prospective studies evaluating the utility of platelet counts in patients must continue being carried to inform evidence-based medical practice.

## References

[1] CARE case report guidelines.

[2] Madziyire MG, Magwali TL, Gidiri MF (2016). A review of malaria in pregnancy. Cent Afr J Med.

[3] Schantz-Dunn J, Nour NM (2009). Malaria and pregnancy: a global health perspective. Rev Obstet Gynecol.

[4] Munjanja SP (2007). Ministry of Health and Child Welfare Zimbabwe maternal and perinatal mortality study 2007.

[5] Dube B, Mberikunashe J, Dhliwayo P, Tangwena A, Shambira G, Chimusoro A (2019). How far is the journey before malaria is knocked out malaria in Zimbabwe: results of the malaria indicator survey 2016. Malar J.

[6] Yagnik K, Farooqi B, Mandernach MW, Cannella AP, Kalyatanda G (2019). Severe case of plasmodium falciparum malaria in a pregnant woman from Nigeria. Case Rep Infect Dis.

[7] Lopez-Perez M, Pacheco MA, Buriticá L, Escalante AA, Herrera S, Arévalo-Herrera M (2016). Malaria in pregnancy: a passive surveillance study of pregnant women in low transmission areas of Colombia, Latin America. Malar J.

[8] Seal SL, Mukhopadhay S, Ganguly RP (2010). Malaria in pregnancy. J Indian Med Assoc.

[9] Pineros JG, Tobon-Castano A, Alvarez G, Portilla C, Blair S (2013). Maternal clinical findings in malaria in pregnancy in a region of North-Western Colombia. Am J Trop Med Hyg.

[10] Dorman E, Shulman C (2000). Malaria in pregnancy. Curr Obstet Gynaecol.

[11] Madziyire G, Magure T, Mandozana G, Mberikunashe J (2017). Uptake of intermittent preventive therapy in pregnancy for malaria in Zimbabwe 2013-2015 Audit. Cent Afr J Med.

[12] Ahmed R, Poespoprodjo JR, Syafruddin D, Khairallah C, Pace C, Lukito T (2019). Efficacy and safety of intermittent preventive treatment and intermittent screening and treatment versus single screening and treatment with dihydroartemisinin-piperaquine for the control of malaria in pregnancy in Indonesia: a cluster-randomised, open-label, superiority trial. Lancet Infect Dis.

[13] Akinosoglou KS, Solomou EE, Gogos CA (2012). Malaria: a haematological disease. Hematology.

[14] Mutala AH, Badu K, Owusu C, Agordzo SK, Tweneboah A, Abbas DA (2020). Impact of malaria on haematological parameters of urban, peri-urban and rural residents in the Ashanti Region of Ghana: a cross-sectional study. AAS Open Res.

[15] Lacerda MVG, Mourão MPG, Coelho HC, Santos JB (2011). Thrombocytopenia in malaria: who cares. Mem Inst Oswaldo Cruz.

[16] Laochan N, Zaloumis SG, Imwong M, Lek-Uthai U, Brockman A, Sriprawat K (2015). Intervals to plasmodium falciparum recurrence after anti-malarial treatment in pregnancy: a longitudinal prospective cohort. Malar J.

[17] Nambozi M, Kabuya JB, Hachizovu S, Mwakazanga D, Mulenga J, Kasongo W (2017). Artemisinin-based combination therapy in pregnant women in Zambia: efficacy, safety and risk of recurrent malaria. Malar J.

[18] Hobeika EM, Usta IM, Taher AT, Nassar AH (2005). Splenomegaly, pancytopaenia and pregnancy: a case report and review of the literature. J Infect.

[19] Bergmann F, Rath W (2015). The differential diagnosis of thrombocytopenia in pregnancy-an interdisciplinary challenge. Dtsch Arztebl Int.

[20] Adams TM, Allaf MB, Vintzileos AM (2013). Maternal thrombocytopenia in pregnancy: diagnosis and management. Clin Lab Med.

[21] Awoke N, Arota A (2019). Profiles of hematological parameters in plasmodium falciparum and plasmodium vivax malaria patients attending tercha general hospital, Dawuro zone, south Ethiopia. Infect Drug Resist.

[22] Nyanga JLN, Wolloh GCM, Yunga NB, Jacqueline N, Ndamukong NC (2020). Malaria parasitaemia and variations in haematological parameters among pregnant women in Buea. Int J Pathog Res.

[23] Sakzabre D, Asiamah EA, Akorsu EE, Abaka-Yawson A, Dika ND, Kwasie DA (2020). Haematological profile of adults with malaria parasitaemia visiting the Volta Regional Hospital, Ghana. Adv Hematol.

[24] Khan SJ, Abbass Y, Marwat MA (2012). Thrombocytopenia as an indicator of malaria in adult population. Malar Res Treat.

[25] Hanson J, Phu NH, Hasan MU, Charunwatthana P, Plewes K, Maude RJ (2015). The clinical implications of thrombocytopenia in adults with severe falciparum malaria: a retrospective analysis. BMC Med.

[26] Tan SO, McGready R, Zwang J, Pimanpanarak M, Sriprawat K, Thwai KL (2008). Thrombocytopaenia in pregnant women with malaria on the Thai-Burmese border. Malar J.

